# On the Progress and Present State of the Practice of Vaccination

**Published:** 1811-11

**Authors:** T. Bateman

**Affiliations:** Bedford Row


*For the Medical and Physical Journal.*

Dear Doctor,

You were probably very much surprised (though you could not be more so than myself) on seeing a communication from me, in the last number of the *new* Journal, edited by your predecessor, Dr. Shearman. You will agree with me, that there is something very disingenuous in reprinting a paper from another periodical work, not only without acknowledgment, but interpolating at the same time the little words, words, "*I am, &c.*" before the signature, in order to deceive the reader into the belief, that such a communication had been made directly by the writer. The fact is, I drew up this summary of the latest official Reports relative to vaccination, at the suggestion of a friend, for insertion in a work, published for charitable purposes, entitled the Philanthropist; and it was not, of course, intended for the perusal of Medical readers. From this work it has been copied, with the sly interpolation before mentioned; and I now send the original to you, chiefly with the view of having this opportunity of explaining to you the circumstance of its unexpected appearance.

I am, Yours sincerely,

T. BATEMAN.

*Bedford Row, Oct.* 19, 1811.

*To Dr. Fothergill.*

---

### *On the Progress and present State of the Practice of Vaccination.*

THE objects, which the general adoption of vaccine inoculation will accomplish for mankind, if time and experience shall confirm the promises of its benevolent discoverer, are so important, that every friend of humanity must have followed, with anxious hope, the progress of the practice, and rejoiced at the general result of the evidence in its favour. It is not easy, indeed, to calculate the sum of human misery that will cease to exist, when the prospect, which vaccination holds out to us, shall be realized. In its casual, or *natural* occurrence, as it is termed, the small-pox is not only the most loathsome distemper that visits the human frame, but the most fatal pestilence: sweeping off multitudes, during its prevalence, and destroying the sight, corrupting the habit, or otherwise inflicting disease, on great numbers of those, who escape its more destructive effects. The practice of inoculation had, it is true, already diminished those evils, among the individuals who resorted to it; but it had unfortunately augmented the evils, among the people in general, by the perpetual infection which it disseminated, and the artificial epidemic which it constantly kept up. In London, for instance, during the first thirty years of the eighteenth century, before inoculation could yet have had any effect, the proportionate number of deaths occasioned by small-pox, as stated in the bills of mortality, was about seventy-four out of every thousand: but, during an equal number of years at the end of the century, the number amounted to nearly one-tenth of the whole mortality, or ninety-five out of every thousand.

So that, as far as we are able to judge from hence, the practice of inoculation, which in itself might be esteemed one of the greatest improvements ever introduced into the medical art, has actually multiplied the ravages of the disease, which it was intended to ameliorate, in the proportion of above five to four.* And the extent of the mischief inflicted on the survivors is manifest from a statement, published by the Society for teaching the Indigent Blind, that nearly one-fourth of the persons admitted into that Charity have been deprived of their sight by the small-pox; not to mention the various forms of scrofula, and other diseases, which it frequently excites.

It is true, that the more intelligent classes of society, who have generally adopted the practice of inoculation, have in a considerable degree avoided the worst of these consequences of small-pox: they have seldom been deprived of the blessing of sight; and they have only been destroyed by the disease in the proportion of about one in three hundred. But the humane will shudder at the recollection, that this exemption has been obtained at the expense of so much additional misery inflicted on the people at large; and that they have but shifted a part of the evils from themselves, to be aggravated in the families of their less enlightened neighbours; while they perpetuate a plague, which would otherwise have had its periods of absolute cessation.

Such is the condition in which the most *improved* state of the art of medicine had placed us, before the benefits of vaccination were discovered; and such is the condition, to which some persons would advise us to return, in consequence of the alleged insecurity of this preventive. But it would seem to be only necessary to take a clear and dispassionate view of the state of the facts, relative to the efficacy of the cow-pox, up to the present time, in order to be convinced of its incalculable advantages, even were all the reported failures proved to have occurred,—nay, if they had actually occurred to double the extent that has been represented. It is the purport of this paper to detail, in as brief a manner as possible, the sum of the facts which have recently been brought to light, and to point out the inference, which seems to be justly deducible from them.

The National Vaccine Establishment, supported by Parliament, has published two Reports during the present year, containing the evidence which they have collected from various

* See the Tables drawn up by Dr. Heberden, in his "Observations on the Increase and Decrease of different Diseases, &c." p. 36.

rious authentic sources. The Colleges of Physicians and Surgeons at Edinburgh, and the Faculty of Glasgow, have again given their decided testimony in favour of vaccination. They assert unanimously, that the practice of vaccination is generally approved of by the profession throughout Scotland; that no bad effects can be ascribed to the practice; and that, since its introduction into Scotland, the mortality occasioned by small-pox has very greatly decreased. The Faculty of Physicians and Surgeons of Glasgow further state, that, since the middle of May, 1801, they have gratuitously vaccinated in their Hall 14,500 persons; and that, as far as is known, the "vaccination in all these has succeeded."*

The accounts from several public Institutions, in and near London, are equally favourable.† In the Royal Military Asylum for the children of soldiers, where between eleven and twelve hundred are now received, vaccination has been practised since its first establishment in the year 1803. From that period to the present time, but *one* instance of death from small-pox has occurred; and, it is worthy of remark, that the individual had not been vaccinated, in consequence of a declaration of the mother, that he had passed through the small-pox in his infancy. Vaccination was introduced into the Foundling Hospital in the year 1801, and every infant, soon after its admission, has since that period been vaccinated. From the commencement of this practice to the present time, no death has occurred from small-pox, and in no instance has the preventive power of vaccination been discredited, although many children, as a test of its efficacy, have been repeatedly inoculated with the matter of small-pox, and exposed to the influence of its contagion. A similar success has attended the practice of vaccination at the Lying-in Charity of Manchester, where, in the space of nine years, more than nine thousand persons have been effectually vaccinated,

---

* Report from the Vaccine Establishment, 1811.

† 1811.

It appears, that since the last Annual Report of the London Vaccine Institution, there have been inoculated by by Dr. Walker . . . . . 2,490
From the commencement of the Institution in 1806 . 8,595
By the appointed Inoculators in the metropolis last year 1,046
From the beginning . . . . . 3,109
By the appointed Inoculators in the country . . 20,801
From the beginning . . . . . 177,474
Last year, Charges of Matter . 31,992 to 6,539 Applicants.
From the commencement of the Institution, 93,080 to 18,900 Applicants. Editor of the Philanthro.

and and secured from the small-pox. The officers of the Vaccine Establishment in London, through the medium of their correspondence with many similar Establishments in the country, have learned, that practitioners of the highest respectability are earnestly engaged in promoting the extension of the practice; that, among the superior classes of the people, vaccination is every where generally adopted; and that, although the prejudices of the lower orders, which have been excited by interested persons, still exist, they appear to be gradually yielding to a conviction of its benefits. This inference is likewise confirmed by the fact, that 23,362 charges of vaccine matter have been distributed by the Establishment to various applicants from all parts of the kingdom, which exceeds by nearly one-third the number distributed in the preceding year.

Of the immense benefits resulting from the universal adoption of vaccination in other countries, the accounts from India have furnished the most interesting example. The number vaccinated in the island of Ceylon, from the year 1802 to January 1810, amounts to no less than 128,732 persons; and, the small-pox has literally been exterminated from the island. From the month of February 1808, to the last mentioned date, the disease had not existed in any part of the island, except in October 1809, when it was carried thither by a boat from the Malabar coast: but, in this instance, the contagion spread to only six individuals, who had not been vaccinated, and was immediately arrested in its progress, and disappeared. The medical Superintendant General observes, that they have no apprehension that the small-pox will ever spread epidemically in Ceylon, while vaccination continues to be generally practised; at the same time, that its occasional appearance there has the good effect of proving the preservative power of the vaccine pock, and of rouzing the natives from their apathy on the subject. Even the Brahmins are now surmounting the prejudices of their education, and submitting to be vaccinated.*

It appears from a Report of the Central Committee of the Vaccine Institution, at Paris, published on the tenth anniversary of its establishment, that the benefits of vaccination, in augmenting the population of a country, have not escaped the attention of the present ruler of France, who has formed depots of vaccine fluid in twenty-four of the principal cities, communicating with the Central Committee, at Paris. In some of the departments, it is said, the zeal of the prefects

* See the Report of the Vaccine Establishment.

has been such, that there remain none to vaccinate, but the infants born in every year, and that the small-pox is already unknown. And the returns of the mortality in the city of Paris, for the year 1809, exhibit only 213 deaths by small-pox. "This number," say the reporters, "though yet too considerable, since the vaccine offered to these 213 victims a certain method of preservation, is yet extremely small in comparison of that of some years, when the epidemic small-pox has carried off, in the same city, more than 20,000 individuals." The Committee, consisting of sixteen of the principal physicians of Paris, express their conviction of the efficacy of vaccination in these terms. "Ten years of labour and success have at length decided the important question, as to the vaccine possessing the power of preserving all those, in whom it has regularly gone through its progress, from the small-pox. This has been carried to such a degree of certainty by the experiments of the Central Committee, and its numerous correspondents, as well Frenchmen as strangers, that there is not at present any fact in medicine better proved, or more certain, than that which establishes the truly *anti-variolous* power of the vaccine."*

Such is the result of the progressive experience of professional men, in regard to the efficacy and preventive powers of vaccination: such is the confirmation, which the inferences, drawn from the early investigation of this subject, have received from subsequent and more extensive research! Insomuch that the conclusion of the College of Physicians upon the subject, in the year 1807, must now be deemed indisputable, that "the truth seems to be established as firmly as the nature of such a question admits."†

The opposition to the practice, which is still but too successfully kept up by a few clamorous individuals in the medical profession, rests principally upon a mistaken view of the nature of the question. It rests upon the notion that the result of the practice should be uniform and invariable,—that the *rule* should be void of all *exceptions*. But there is no such regularity in the operations of the animal economy; there is no disease without its anomalies; and the diversity of human constitutions is infinite. Several of those anomalies, or exceptions to the general rule, have doubtless occurred in the practice of vaccination; "but" to use the words of a judi-

* A copy of this Report may be found in the Edinburgh Med. and Surg. Journal, for Jan. 1811, p. 117.

† See the Report of the Royal College of Physicians on Vaccination, July 1807.

cious cious and experienced observer, "certainly not so often as was expected by those, who considered the subject from the first dispassionately, nor have they been in sufficient number to form any serious objection to the practice founded on Dr. Jenner's discovery."* In truth, if this principle were received, that no operation ought to be performed on the human body which was liable to occasional failure, what medicine would remain for us to exhibit, or what surgical assistance for us to offer?

But let us examine the nature of these exceptions, or "failures," as they have been emphatically called, which have occurred in the practice of vaccination. The very sound of the word excites an alarm, in the minds of many persons, as if *failure* were synonimous with *death*, or implied the certain occurrence of a desperate or mortal small-pox. But this is so far from being the case, that upon a deliberate view of the facts, we do not hesitate to affirm, that, if all the cases of alleged failure, which the opponents of vaccination have raked up, upon any sort of evidence, and often upon none, had really occurred, and that number had been doubled or tripled, its advantages over the inoculation of small-pox would still be incalculable.

In the first place, it has been ascertained by the concurring observations of almost all the practitioners who have attended to the subject, that (to use the words of the College of Physicians) "in almost every case in which the small-pox has succeeded vaccination, whether by inoculation or by casual infection, the disease has varied much from its ordinary course; it has neither been the same in *violence*, nor in the *duration* of its symptoms, but has, with very few exceptions, been *remarkably mild, as if the small-pox had been deprived by the previous vaccine disease of its usual malignity*."† Dr. Willan states, that the feverishness, which precedes the eruption in these cases, is often considerable, but the pustules are small and hard, containing little or no matter, and begin to dry off on the sixth day.‡ It must not be omitted, indeed, that, in a very few instances, the small-pox, subsequent to vaccination, has assumed the confluent form, and put on a dangerous aspect (as in the recent case of the son of Earl Grosvenor); but even in these rare instances, the modifying influence of the previous vaccination has been manifest, the disease, when near its height, receiving a sudden check, and the recovery being

---

* See Dr. Willan's Treatise on Vaccination, p. 21.

† See the Report of the College.

‡ See his Treatise, Sect. iv.

unusually rapid.* One case of this sort occurred to the observation of the writer of this paper, in which on the seventh day of confluent small-pox, the child became suddenly free from constitutional complaint, and ran about at play;—a circumstance, he believes, that is never known to occur in confluent small-pox, where the previous influence of vaccination had not been exerted. In this statement, then, we have admitted the worst consequences that have ever accompanied the "failures" of vaccination, in any one instance.

But, in the second place, let us attend to the proportionate number of these failures. "It does not appear," says Dr. Willan, who minuted the cases as they happened, "that failures in the preventive effect of vaccine inoculation, including *mistakes*, *negligences*, and *mis-statements*, have occurred in a greater proportion than as *one* to *eight hundred*.†" It is very improbable, then, that the actual failures amount to one in a thousand, or to any thing near that number. But let us suppose, for the sake of argument, that the failures amount to the proportion of one in five hundred; that is to say, that one of every five hundred persons vaccinated, remains liable to be infected by small-pox: and let us further imagine that this subsequent small-pox is not mitigated in any case, and therefore, that (as in the case of the ordinary *natural* small-pox) *one* in *six* of these will die. Then the worst result would be, that *one*, out of every *three thousand* persons vaccinated, would die. But we know, that *one* of *three hundred* persons, who receive the small-pox by inoculation, perishes of that disease.‡ The conclusion is therefore obvious, that the worst result that could be calculated upon from vaccine failures, would leave the balance in favour of vaccination, in the proportion of *ten* to *one*. But, when we consider the actual state of the circumstances;—that the number of deaths from inoculated small-pox really exceeds the number of "failures" of vaccination;—that these "failures" are, in a great majority of instances, the means of insuring a very mitigated and harmless small-pox;—and that they have, perhaps, in no instance, been followed by a fatal small-pox;—the chances of fatality from a failure of the vaccination are so trivial as to elude calculation, and the only chance of in-

* See his last Report of the National Vaccine Establishment, July, 1811.

† See his Treatise, p. 23.

‡ Doctor Willan states that "the inoculated small-pox still proves fatal in *one* case out of *two hundred and fifty*."—*Ibid.*

jury jury that ensues, is reduced to that of a temporary inconvenience.

Lastly, let us reflect on the non-contagious nature of the vaccine disease, which, while it secures the individual from blindness, deformity or fatality, too often consequent on the small-pox, injures no one, and spreads no epidemic around, and we shall be compelled to admit, that, "with all its imperfections on its head," with a frequency of failure that its most active opponents have never yet ascribed to it, vaccination would still prove a blessing, such as few individuals have had the happiness to confer upon mankind.

We might here have terminated our observations, but the leading circumstance, communicated in the late Report from the National Vaccine Establishment, demands some notice. It is singular, that at the time when the public attention was attracted by the occurrence of small-pox, after vaccination, in the sons of the Earl of Grosvenor and Sir Henry Martin, the second occurrence of small-pox in the Rev. Joshua Rowley, Miss Booth, and two other persons, should have happened.* In three of these cases, the previous small-pox had been taken by inoculation, and in the fourth, in the natural way. But the truth is, that the small-pox itself, in whichsoever of these two ways it is produced, is liable to the same anomalies and exceptions as the cow-pock. There are several examples of the fact on record; one of the most striking of which is the case of Mr. Langford, related in the 4th volume of the Memoirs of the Medical Society of London. This person was so "remarkably pitted and seamed" by a former malignant small-pox, "as to attract the notice of all who saw him:" yet he died at the age of fifty, in an attack of confluent small-pox, in which he communicated the infection to five other individuals of the family, one of whom also died. It will be unnecessary here to detail the various examples which authors have described. The writer will just notice an instance, which occurred under his own observation not long ago, the particulars of which will be detailed in the 2d volume of the "Medico-chirurgical Transactions," about to be published.† This occurred in a woman, of twenty-five years of age, who was considerably pitted by a former confluent small-pox, which she had suffered in her childhood. She caught the second disease, which went through the usual variolous stages in a mild way, by nursing

* See the Report of July 1811.

† Several cases and many references will be there found, which are omitted here for the sake of brevity.

her her infant under a confluent small-pox, which proved fatal to it. It is remarkable, that her two elder children, who had been vaccinated a few years before, lived in the same apartment during the progress of the small-pox in the infant and mother, and escaped the infection; the cow-pock in them having exerted a preventive power, which the previous small-pox had failed to effect in the mother. The poor woman had been prevented, by the terrors excited by the anti-vaccinists, from vaccinating her youngest child: a fact which should induce these opponents of the practice to reflect on the serious responsibility which they assume, in thus discouraging the adoption of this important preventive.

T. BATEMAN.

*Bedford Row, August* 19, 1811.

